# Resveratrol Attenuates 2,3,7,8-Tetrachlorodibenzo-p-dioxin-Mediated Induction of Myeloid-Derived Suppressor Cells (MDSC) and Their Functions

**DOI:** 10.3390/nu15214667

**Published:** 2023-11-03

**Authors:** Wurood Hantoosh Neamah, Alex Rutkovsky, Osama Abdullah, Kiesha Wilson, Ryan Bloomquist, Prakash Nagarkatti, Mitzi Nagarkatti

**Affiliations:** Department of Pathology, Microbiology and Immunology, University of South Carolina School of Medicine, Columbia, SC 29228, USA; wurood.neamah@uobasrah.edu.iq (W.H.N.); acrutkovsky@gmail.com (A.R.); osama.abdulla@uomosul.edu.iq (O.A.); kiesha.wilson@uscmed.sc.edu (K.W.); ryan.bloomquist@uscmed.sc.edu (R.B.); prakash@mailbox.sc.edu (P.N.)

**Keywords:** TCDD, dioxin, resveratrol, immunosuppression, MDSC, neutrophil, AhR

## Abstract

Previously, we showed that 2,3,7,8-Tetrachlorodibenzo-p-dioxin (TCDD), an aryl hydrocarbon receptor (AhR) ligand and a potent and persistent toxicant and carcinogenic agent, induces high levels of murine myeloid-derived suppressor cell (MDSC) when injected into mice. In the current study, we demonstrate that Resveratrol (3,4,5-trihydroxy-trans-stilbene; RSV), an AhR antagonist, reduces TCDD-mediated MDSC induction. RSV decreased the number of MDSCs induced by TCDD in mice but also mitigated the immunosuppressive function of TCDD-induced MDSCs. TCDD caused a decrease in F4/80+ macrophages and an increase in CD11C+ dendritic cells, while RSV reversed these effects. TCDD caused upregulation in CXCR2, a critical molecule involved in TCDD-mediated induction of MDSCs, and Arginase-1 (ARG-1), involved in the immunosuppressive functions of MDSCs, while RSV reversed this effect. Transcriptome analysis of Gr1^+^ MDSCs showed an increased gene expression profile involved in the metabolic pathways in mice exposed to TCDD while RSV-treated mice showed a decrease in such pathways. The bio-energetic profile of these cells showed that RSV treatment decreased the energetic demands induced by TCDD. Overall, the data demonstrated that RSV decreased TCDD-induced MDSC induction and function by altering the dynamics of various myeloid cell populations involving their numbers, phenotype, and immunosuppressive potency. Because MDSCs play a critical role in tumor growth and metastasis, our studies also support the potential use of RSV to attenuate the immunosuppressive properties of MDSC.

## 1. Introduction

2,3,7,8-Tetrachlorodibenzo-p-dioxin (TCDD) is a carcinogen and a highly immunosuppressive environmental contaminant [1,2]. TCDD is well established as a potent agonist of aryl hydrocarbon receptor (AhR; AHR), a ligand-activated transcription factor that regulates a variety of cues including environmental, dietary, microbial, and metabolic to control a variety of physiological and immunological functions [3]. AHR is a member of the basic-helix/loop/helix per-Arnt-sim (bHLH/PAS) family of transcription factors. While AhR activation not only serves as an environmental sensor that regulates the effects of environmental toxins such as TCDD, it also acts as a key signaling pathway leading to attenuated inflammation.

Myeloid-derived suppressor cells (MDSCs) represent a heterogeneous population of myeloid cells that are well characterized for their immunosuppressive properties [4]. MDSCs play a critical role in suppressing antitumor immune responses mediated by T and natural killer (NK) cells and thereby promote tumor growth [5]. MDSCs also constitute a hindrance to successful cancer immunotherapy because of their immunosuppressive properties [4]. MDSCs function through multiple mechanisms including depletion of extracellular arginine, secretion of immunosuppressive mediators and cytokines [6], and reactive oxygen species (ROS) production [7], among others [8].

Interestingly, our laboratory has recently shown that the activation of AhR by TCDD can trigger massive induction of MDSCs that are highly immunosuppressive [9]. TCDD induced the expression of CXC chemokine receptor 2 (CXCR2) and thus, blocking this receptor led to decreased induction of MDSCs. MDSCs consist of two main subsets: granulocytic or polymorphonuclear MDSCs (PMN-MDSCs) defined as CD11b^+^Ly6G^+^Ly6C^low^ cells with high side scatter and monocytic MDSCs (M-MDSCs) defined as CD11b^+^Ly6G^–^Ly6C^hi^ cells with low side scatter [10,11]. TCDD was previously shown by us to induce primarily PMN-MDSCs and to a lesser extent the M-MDSCs [9].

Resveratrol (3,5,4′-trihydroxy-trans-stilbene; RSV) is plant-derived natural polyphenol and a phytoestrogen that has been shown to exhibit anti-inflammatory properties [12,13]. RSV is also known to act as an AhR antagonist [14]. AhR is localized in the cytosol in a complex composed of HSP90, AIP, p23, and c-SRC [14]. When activated by AhR agonists, such as TCDD, conformational changes occur in AhR leading to its translocation to the nucleus. In the nucleus, AhR interacts with ARNT, and the heterodimer is responsible for the transcription of genes that express the dioxin responsive elements (DREs). Interestingly, RSV was shown to promote AhR translocation to the nucleus and binding to DNA at DREs, but it failed to further cause the transactivation [14]. Thus, RSV inhibits the transactivation of several genes that are inducible by TCDD including cytochrome P-450 1A1 [14].

In the current study, we investigated if RSV would attenuate the ability of TCDD to induce MDSCs and alter their immunosuppressive functions. Our goal was to study the nature of gene expression and metabolic changes occurring in MDSCs after TCDD exposure and investigate if RSV would reverse these effects. Our data demonstrate that RSV can indeed reverse the number, gene expression profile, and immunosuppressive functions of MDSCs. These findings support strategies aimed at altering disease outcomes from TCDD exposure using RSV to influence immune modulation of MDSC generation and function. 

## 2. Methods 

### 2.1. Experimental Animals

Female 6–8-week-old C57BL/6 mice were purchased from Jackson Laboratory, Bar Harbor, ME, USA and housed in specific pathogen-free conditions at the AAALAC-accredited University of South Carolina, School of Medicine, Animal Resource Facility, for two weeks prior to use. All experiments were approved by the Institutional Animal Care and Use Committee (IACUC) at The University of South Carolina. There are sex differences in the toxicity induced by TCDD [15]. Also, resveratrol is considered to be a phytoestrogen [16]. It is for that reason, we did not mix male and female mice and used only female mice. 

### 2.2. Chemicals and Reagents

The 3,4,5-trihydroxy-trans-stilbene (resveratrol, RSV) was purchased from Sigma-Aldrich, St. Louis, MO, USA and 2,3,7,8-Tetrachlorodibenzo-p-dioxin (TCDD) was a kind gift from Dr. Steve Safe (Institute of Biosciences & Technology, Houston, TX, USA; A&M Health Sciences Center, College Station, TX, USA). Culture medium and buffer reagents (RPMI 1640, Penicillin-Streptomycin, HEPES, L-Glutamine, EDTA, FBS, and PBS) were purchased from Invitrogen Life Technologies (Carlsbad, CA, USA). The antibodies used for surface marker staining or selection were purchased from BioLegend (San Diego, CA, USA): Alexa Fluor 700 (clone M1/70; 101222) or FITC-conjugated anti-Cd11b (clone M1/70; 101206), BV510 (clone RB6-8C5; 108457) or PE-conjugated anti-Gr1 (clone RB6-8C5; 108408), BV421-conjugated anti-F4/80 (clone BM8; 123137) or PE-conjugated anti-F4/80 (clone BM8; 123110), Alexa Fluor 488 (clone HK1.4; 128021) or BV650-conjugated anti-Ly6C (clone HK1.4; 128049), BV785 (clone 1A8; 127645) or PE-conjugated anti-Ly6G (clone 1A8; 127608), PerCPCy5 (clone M5/114.15.2; 746197) or BV421-conjugated anti-MHCII (clone M5/114.15.2; 107632), Alexa Fluor 647-conjugated anti-cKit (clone 2B8; 105817), PE-CF594-conjugated anti-Fcer1 (clone MAR-1; 134307), and BV605 (clone N418; 117334) or PE-conjugated anti-Cd11c (clone N418; 117307). The TruStain FcX Block reagent (anti-mouse CD16/32; clone 93; 101320) was procured from BioLegend and PE Positive Selection Kits were purchased from StemCell Technologies (Vancouver, Canada). Zombie Aqua Fixable Viability Kit (423102) was purchased from BioLegend. The XFp glycolytic rate assay kit was purchased from Agilent Technologies (Santa Clara, CA, USA). 

### 2.3. Treatment Regime

We used four groups of mice in this study: (1) Naïve, (2) RSV, (3) TCDD, and (4) TCDD+RSV. In the TCDD+RSV group, C57BL6 mice received RSV at a dose of 50 mg/kg dissolved freshly in 1% carboxymethyl cellulose (CMC) solution and administered by oral gavage. Twenty four hours later, these mice received TCDD dissolved in corn oil by the intraperitoneal route at a dose of 10 µg/kg, as described previously [9]. Ninety minutes later, these mice received a second dose of RSV followed by a dose of 50 mg/kg through the oral route. Twenty-four hours after TCDD treatment, the mice were euthanized for studies on MDSCs. TCDD group received 10 µg/kg of TCDD along with the vehicle used for RSV and the RSV group received 50 mg/kg of RSV as described above along with vehicle used for TCDD. Additionally, the Naïve mice orally received the vehicle used for RSV (1% CMC) and the vehicle for TCDD (corn oil).

### 2.4. Flow Cytometry Staining for Cell Subtypes

Peritoneal exudate cells were harvested as described [9], and one million cells were stained for flow cytometry using a BD FACs Celeste. Individually stained populations or UltraComp eBeads (Invitrogen, Eugene, OR, USA) were used for compensation. Flow cytometric analysis and t-distributed stochastic neighbor embedding (tSNE) generation was performed with FlowJo software version 10.7 (Becton, Dickinson & Company, Franklin Lakes, NJ, USA). An equal number of cells from Zombie live populations per group were used to generate a tSNE plot. 

### 2.5. Strategy to Define Immune Cell Phenotypes

Cells were harvested from the peritoneal cavity of Naïve, RSV, TCDD, and RSV+TCDD mice and stained with Cd11b and Gr1 to identify MDSCs (Cd11b^+^Gr1^+^) and LY6C and LY6G to identify Monocytic (Cd11b^+^Ly6G^−^Ly6C^hi^) and Polymorphonuclear/Granulocytic (Cd11b^+^Ly6G^+^Ly6C^lo^) MDSCs as described previously [17]. According to the gating strategy of Ghosn and Cassado et al. [18], Cd11b and F4/80 was used to define monocytes (Cd11b^+^F4/80^−^). Cd11c and Gr1 markers were used after gating on Cd11b to determine dendritic cells (Cd11c^+^Gr-1^−^). The expression of MHCII was used to detect antigen-presenting cells. 

### 2.6. Purification of MDSCs

MDSCs were purified from the peritoneal exudates of Naïve, RSV, TCDD, and RSV+TCDD mice, as described previously [19]. In brief, peritoneal exudate cells were collected and labeled with PE-conjugated anti-Gr1 antibody. The EasySep Ms PE Positive Slcn Kit II from StemCell Technologies, Vancouver, Canada (17696) was used for selection. After purification, flow cytometry was used to assess the purity of selected cells. 

### 2.7. Transcriptome of Gr1+ Cells

Purified Gr1^+^ cells were analyzed by Affymetrix GeneChip WT PLUS Reagent Kit transcriptome microarray according to manufacturer’s protocols and as previously described on an Affymetrix GCS 3000 system. Briefly, RNA was lysed in Qiazol and purified using the RNeasy Kit (Qiagen, Santa Clara, CA, USA). Signal values of probes were used to determine the fold change between pairwise comparisons. Quality control, data summarization, and normalization were performed with Transcriptome Analysis Console (TAC) software version 4.0.1.36 (Thermo Fisher Scientific, Waltham, MA, USA) and Partek Flow software version 11.0.23.1004 (Partek^®^ Flow^®^) to determine differential gene expression, showing consistent over-representation as reported here using Partek Flow results. 

### 2.8. Partek Flow Analysis

Affymetrix Expression Console Cel files were uploaded, STAR–2.7.3a aligned, and quantified to the annotation model (mm10-Ensembl Transcripts release 101). Gene counts were normalized by counts per million (CPM), add 1.0E-4, log 2.0. Differentially expressed features were identified with the Partek GSA algorithm (FDR step up ≤ 0.05; fold-change ±1.5). 

### 2.9. Immunosuppressive Effects of MDSCs on T Cell Proliferation In Vitro

To examine the suppressive effect of MDSCs on T-cell proliferation, splenocytes (5 × 10^5^) from C57BL/6 naïve mice were cultured in the presence of Concanavalin A (Con-A), a potent T-cell activator [20], (2 µg/mL) together with a 1:0.5 ratio of T cells to MDSCs for 24 h, as previously described [21]. Radioactive ^3^H-thymidine (1 µCi/well) was added to the cell cultures. After 18 h, radioactivity was measured using a liquid-scintillation counter (MicroBeta Trilux; PerkinElmer Life and Analytical Sciences).

### 2.10. Measuring Glycolytic Rate

Proton efflux rate (PER) was measured in 2 × 10^5^ purified Gr1+ cells from the peritoneal cavity of mice using the Seahorse Bioscience XF Extracellular Flux Analyzer (Agilent Technologies) with manufacturer recommended conditions. Briefly, Gr1-selected cells were plated in the XF cell culture plate coated with 15 μg CellTak in phenol red-free complete Medium enriched with 2 mM glutamine, 10 mM glucose, 1 mM pyruvate, and 5 mM HEPES. Cell stress was monitored in response to the addition 0.5 µM Rotenone plus Antimycin A (Rot/AA) and 50 mM 2-Deoxy-D-glucose (2-DG). 

### 2.11. Statistical Analysis

GraphPad Prism software version 9.0.1 (San Diego, CA, USA) was used for statistical analysis with the methods present in the figure legends. A standard *t*-test with Holm–Sidak’s multiple comparisons corrections was used when comparing two groups for significance. A one-way or two-way analysis of variance (ANOVA) with post hoc Tukey’s multiple comparisons test was used to compare more than two groups. Error bars are expressed as mean ± the standard error of mean (±SEM) or mean ± standard deviation of mean (±SD) as indicated in figure legends. Significance was defined as a *p*-value less than 0.05. 

## 3. Results

### 3.1. RSV Attenuated MDSC Accumulation in The Peritoneal Cavity

**Figure 1:**
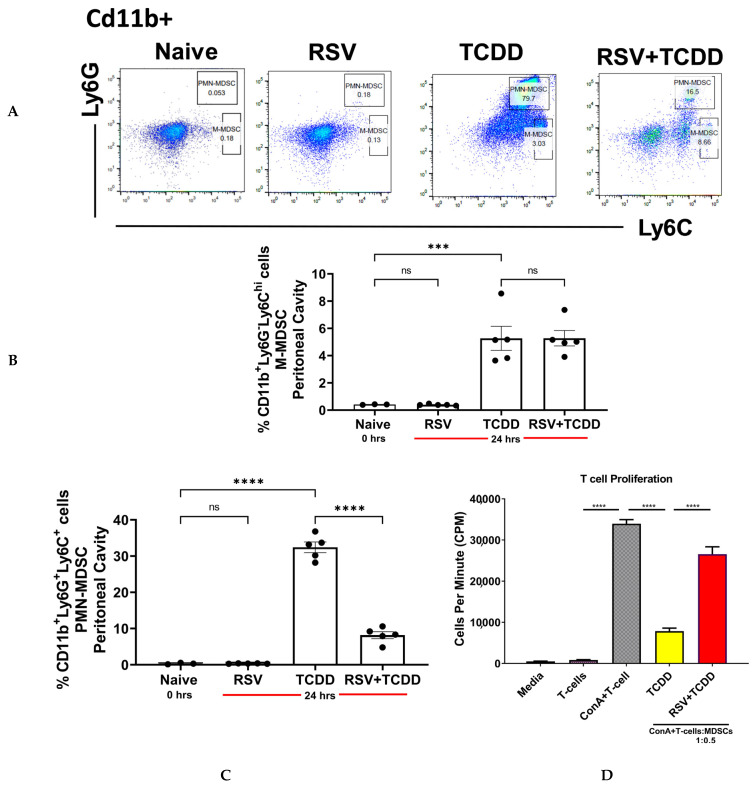
Resveratrol (RSV) attenuates MDSC induction and function by TCDD: Peritoneal exudate cells isolated from various groups of mice were stained to identify MDSCs subsets and function. (**A**) Representative flow cytometric plots showing the percentages of cells gated for CD11b+ cells double-stained for Ly6C and Ly6G. (**B**) Data from the cells obtained from the peritoneal cavity of mice showing the proportion of M-MDSCs (CD11b+Ly6ChiLy6G-). (**C**) Similar data showing the proportion of polymorphonuclear PMN-MDSCs (CD11b+Ly6C+Ly6G+). (**D**) Purified peritoneal MDSCs from TCDD and RSV+TCDD were incubated with spleen cells activated with ConA at a ratio of 1:0.5 (T cells:MDSCs). The vertical bars represent the controls, showing splenic T cells incubated medium (Medium), splenic T cells incubated with ConA (T-cells), splenic T cells activated with ConA and incubated with MDSC from TCDD-treated mice (TCDD), and splenic T cells activated with ConA and incubated with MDSC from RSV+TCDD treated mice. T-cell proliferation was assessed by ^3^H-thymidine incorporation assay. Vertical bars represent mean  ±  SEM of 4 mice per group. One-way ANOVA with Tukey’s multiple comparisons test was performed for statistical significance between comparisons (*** *p*  <  0.001; **** *p*  <  0.0001; ns = not significant).

We previously reported that administration of TCDD leads to activation of AhR and induction of a large number of MDSCs [9]. In contrast, RSV has been shown to act as a competitive antagonist of dioxins at the AhR by promoting AhR nuclear translocation and inhibiting dioxin responsive elements [14]. Thus, we investigated if RSV would alter the induction of MDSCs by TCDD. We found that TCDD induced high levels of granulocytic/polymorphonuclear (PMN)-MDSCs (CD11b+Ly-6G+Ly-6C^low^) and lower levels of monocytic MDSCs (CD11b+Ly6G−Ly-6C^hi^) (Figure 1A) consistent with previous studies [11]. Interestingly, while TCDD was a potent inducer of both MDSC types of MDSCs, RSV treatment greatly attenuated TCDD-mediated induction of PMN-MDSCs but not M-MDSCs, suggesting a lineage specific restriction of RSV on MDSC recruitment (Figure 1B,C). To assess the immunosuppressive function of MDSCs, we cultured T-cells with ConA in the presence of MDSCs derived from TCDD-treated or RSV+TCDD-treated mice and found that while MDSCs derived from TCDD-treated mice greatly decreased T-cell proliferation, RSV partially rescued the inhibitory function of TCDD on proliferation (Figure 1D). Together, these data suggested that RSV was able to reverse the ability of TCDD to induce PMN-MDSCs specifically and block the inhibitory functions of TCDD on T-cell proliferation. 

### 3.2. Effect of TCDD and RSV on Peritoneal Exudate Cells

**Figure 2:**
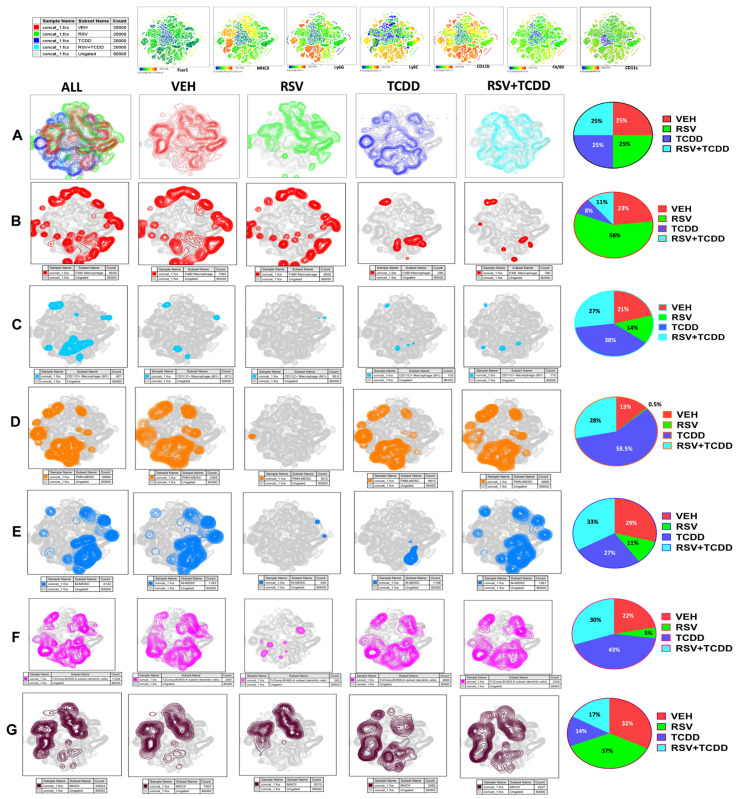
Effect of TCDD and RSV on cells found in the peritoneal cavity. The following groups of mice including Naïve (VEH), RSV, TCDD and TCDD+RSV were studied for characterizing cells from the peritoneal cavity using flow cytometry. The top panel shows the cells stained for various markers. (**A**) Peritoneal exudate cells were stained for myeloid markers and analyzed by flow cytometry. To visualize differences among Zombie live populations, a tSNE plot was generated with 20,000 cells per group excluding scatter. Differences among groups are shown by colors (VEH is red, RSV is green, TCDD is blue, and RSV+TCDD is turquoise). (**B**) Expression of F4/80+ macrophages with the proportions of cells shown in pie chart. (**C**) Expression of CD11c+ macrophages with the proportions of cells shown in pie chart. (**D**) Expression of CD11b+Ly6G+Ly6C+ PMN-MDSCs with the proportions of cells shown in pie chart. (**E**) Expression of CD11b+Ly6G-Ly6Chi M-MDSCs with the proportions of cells shown in pie chart. (**F**) Differential expression of dendritic cells with the proportions of cells shown in pie chart. (**G**) Differential expression of MHCII expression with the proportions of cells shown in pie chart.

To further understand the effect of TCDD on other immune cells and how RSV could alter this effect, multiparameter flow cytometry was performed and dimension reduction was used to visualize the results using T-Distributed stochastic neighbor embedding (tSNE). Peritoneal exudate cells from Vehicle, RSV, TCDD, and RSV+TCDD groups were examined by flow cytometry and the Zombie live populations were used to generate a tSNE plot using FlowJo to visualize differences (Figure 2A). We looked at a variety of myeloid-lineage determining markers such as Fcer1, MHC Class-II, Ly6G, Ly6C, CD11b, F4/80, and CD11c. Relative expression differences among myeloid markers within the clusters are shown (Figure 2A). Because tSNE plot visual density does not accurately reflect the proportion of cells, we also depicted the data as a pie chart for each of the cell populations delineating the effect of TCDD and RSV.

The F4/80 macrophage marker was reduced in the TCDD group when compared to the VEH control, while the RSV+TCDD group showed a slight increase when compared to the TCDD group. Interestingly, RSV alone caused a significant increase in F4/80 macrophages (Figure 2B). This suggests that TCDD was a potent inhibitor of F4/80 macrophages even in the presence of RSV. 

We next studied the CD11c marker which is expressed primarily on dendritic cells but also on M1 macrophages. The expression of the CD11C+ marker was increased in the TCDD group while RES+TCDD showed significant decrease (Figure 2C). Studies on PMN MDSCs revealed that TCDD triggered a significant increase in these cells, similar to the earlier observation (Figure 1), while RSV+TCDD showed significant decrease (Figure 2D). On the other hand, the M-MDSCs were not significantly altered in the TCDD group when compared to the Vehicle group, and RSV+TCDD also did not show much effect; however, RSV alone did reduce the number of M-MDSCs (Figure 2E). While some variation may have occurred between the two assays presented in Figure 1B vs. Figure 2E with respect to M-MDSCs, we note that the action of both TCDD as well as its partial rescue through RSV addition occurred mainly in the PMN-MDSC lineage (Figure 1C and Figure 2D). Also, a subset of dendritic cells was upregulated following TCDD treatment when compared to the Vehicle group, while the RSV+TCDD group showed a significant decrease when compared to the TCDD group (Figure 2F). Additionally, RSV alone resulted in a marked decrease in dendritic cells compared to the vehicle. TCDD caused a marked decrease in MHC Class-II-expressing cells, which are found on antigen-presenting cells including monocytes, macrophages, dendritic cells, and B cells, when compared to the vehicle. In contrast, the RSV+TCDD group showed slight recovery of such cells (Figure 2G). 

### 3.3. RSV Alters Gene Expression Changes and Lessens Energetic Demands Induced by TCDD in MDSCs

**Figure 3:**
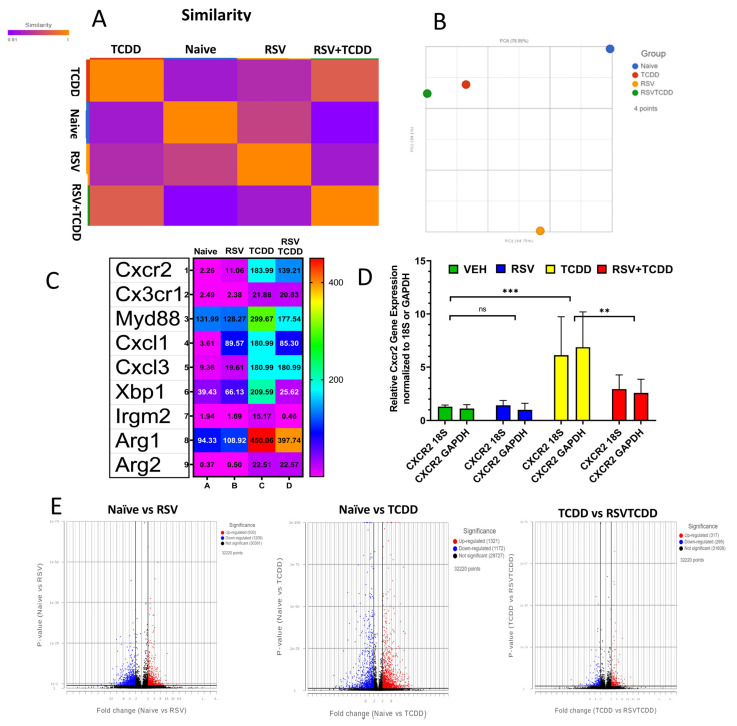
Transcriptome analysis of Gr1+ MDSCs from PC by Affymetrix array (MTA-1.0). Samples were STAR—2.7.3a aligned then quantified to annotation model (Partek E/M) where gene counts were normalized by counts per million (CPM), add 1.0 × 10^−4^, Log 2.0. (**A**) Similarity matrix of samples. (**B**) Principal component analysis (PCA) of samples. (**C**) Heatmap showing gene expression levels of select genes of interest. (**D**) Cxcr2 RT-PCR gene expression levels from at least 3 biological replicates normalized to Vehicle with either 18S or GAPDH as the stable reference gene. An ordinary one-way ANOVA with multiple comparisons of the mean group values (combine 18S and GAPDH) was analyzed and significance is plotted (** *p*  <  0.01; *** *p*  <  0.001; ns = not significant). (**E**) Differentially expressed features were identified with Partek GSA. Volcano plots displaying GSA analysis of comparisons which have been filtered by FDR step up ≤ 0.05.

Because Gr-1 comprises both Ly6C+ and Ly6G+ cells, we next performed transcriptome analysis for Gr-1+ cells in all groups. Partek Flow software was used to analyze the array data and the similarity matrix (Figure 3A) as well as principal component analysis (PCA) (Figure 3B). The PCA of samples showed distinct separation between the 4 groups. Expression changes of key genes known to be important in MDSC recruitment after AhR binding, such as Cxcr2, Cx3cr1, Myd88, Cxcl1, Cxcl3, Xbp1, Irgm2, Arg1, and Arg2, are shown via heatmap with respective values (Figure 3C). These data show that TCDD treatment caused significant induction of Cxcr2, Myd88, Cxcl1, Cxcl3, Xbp1, and Arg1, while the RSV+TCDD group showed reversal of this effect in a majority of the molecules tested. We corroborated these results using RT-PCR for one of the chief markers of TCDD driven MDSC induction, Cxcr2. While TCDD caused a significant increase in Cxcr2, the RSV+TCDD group saw a reduction in this effect (Figure 3D). Additionally, the Volcano plots showed differentially expressed gene trends between the different groups (Figure 3E). These data showed maximum differences between the Naïve vs. TCDD group, while in contrast less differential expression was noted between the RSV+TCDD groups and the vehicle. This finding establishes, in a more comprehensive way, that the TCDD+RSV group shows less perturbation in normal gene expression than TCDD alone, supporting the role of RSV to reverse the effect of TCDD.

### 3.4. Metabolic Pathway Analysis of Cells Exposed to TCDD or RSV+TCDD 

**Figure 4:**
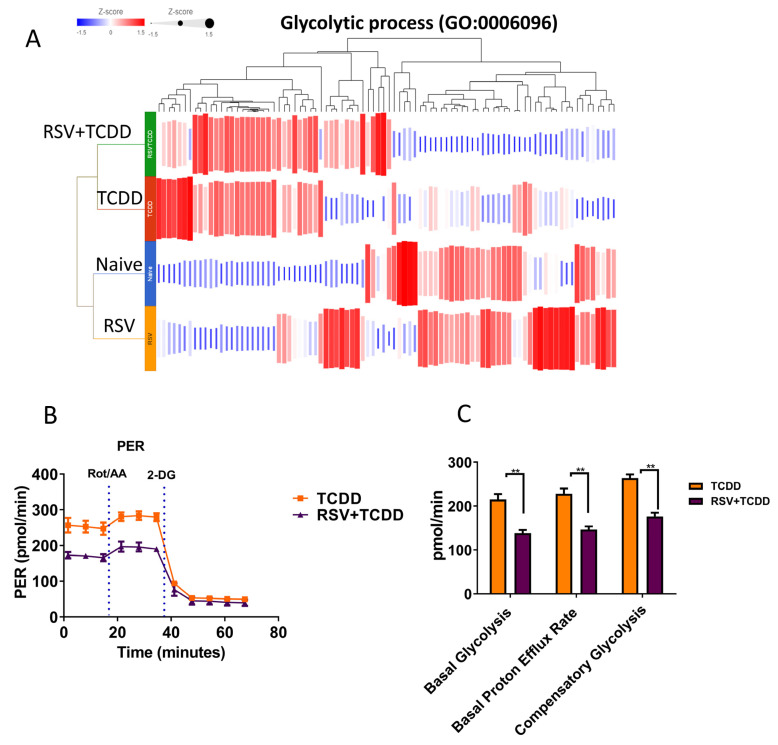
RSV attenuates a TCDD-induced increase in metabolic process genes and altered proton efflux rate (PER) by TCDD in Gr1+ MDSCs. Genes related to Gene Ontology (GO) metabolic pathways were assessed. (**A**) Heatmap showing expression (z-score) of genes per group clustered by cladogram in the glycolytic process (GO:0006096) pathway. (**B**,**C**) Seahorse PER values from TCDD versus RSV+TCDD. Quantification of energetic parameters such as pmol/min, including basal glycolysis (conversion of glucose to lactate), basal proton efflux rate (the number of protons exported by cells over time), and compensatory glycolysis (rate of glycolysis following mitochondrial inhibition of oxidative phosphorylation, forcing use of glycolysis to meet energetic demands) significantly decreased in RSV+TCDD compared to TCDD (** *p*  <  0.01) as analyzed by one-way ANOVA followed by Tukey’s multiple comparisons.

We next investigated if TCDD altered the metabolic pathways in cells and if RSV was able to reverse this effect. A heatmap showing expression (z-score) of genes per group clustered by cladogram in the Glycolytic process (GO:0006096) pathway is shown in Figure 4A. Gene Ontology (GO) analysis showed significant differences in glycolytic pathways between various groups. Overall, TCDD was shown to induce increased expression of genes involved in glycolytic pathways when compared to the Naïve group, while the RSV+TCDD group showed partial reversal of these effects (Figure 4A). Next, we performed the proton efflux rate (PER) using Seahorse in the MDSCs isolated from mice treated with TCDD or TCDD+RSV. The quantification of energetic parameters such as pmol/min, including basal glycolysis (conversion of glucose to lactate), basal proton efflux rate (the number of protons exported by cells over time), and compensatory glycolysis (rate of glycolysis following mitochondrial inhibition of oxidative phosphorylation, forcing use of glycolysis to meet energetic demands) significantly decreased in RSV+TCDD compared to TCDD.

**Figure 5:**
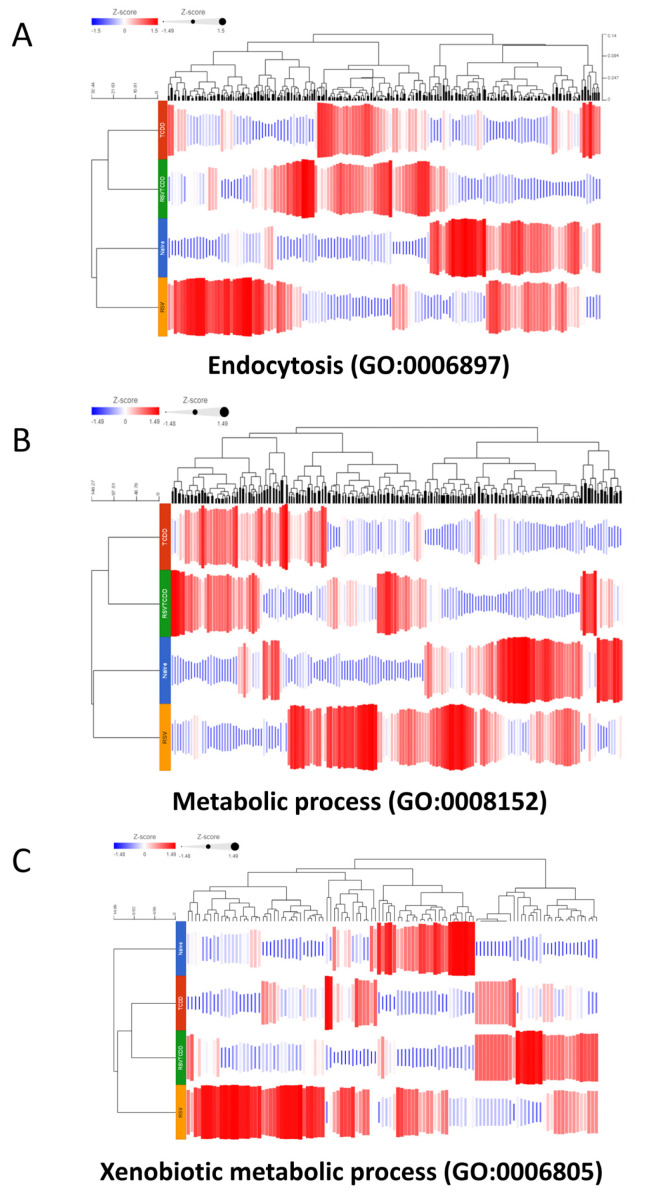
Gene Ontology (GO) and metabolic pathway analysis show differences in endocytosis and other pathways between the groups. Genes related to Gene Ontology (GO) endocytosis, metabolic processes, and xenobiotic metabolic process were assessed. (**A**) The Gene Ontology (GO:006897) pathway related to endocytosis is shown among the groups colored by z-score and grouped according to a cladogram. (**B**) Heatmap showing expression (z-score) of genes per group clustered by cladogram in the metabolic process (GO:0008152) pathway. (**C**) Heatmap showing expression (z-score) of genes per group clustered by cladogram in the xenobiotic metabolic process (GO:0006805) pathway.

Gene Ontology (GO) and endocytosis pathway analysis show differences between the groups. The Gene Ontology (GO:006897) pathway related to endocytosis showed significant variations in the TCDD group when compared to the Naïve group (Figure 5A). The TCDD+RSV group failed to reverse the effects of TCDD to a greater extent. This may be because the RSV group alone showed significant alterations when compared to the Naïve group (Figure 5A). We also tested the genes involved in the metabolic process (GO:0008152) pathway and found that TCDD caused the upregulation in a significant number of genes when compared to the Naïve group, while the TCDD+RSV group showed reversal of some of these genes. When we studied the expression of genes in the Xenobiotic metabolic process (GO:0006805) pathway, TCDD reversed some of the gene expression profiles depicted by the Naïve group while the TCDD+RSV group showed reversal of some of the effects seen in the TCDD group. The RSV alone group also showed a marked increase in the expression of a majority of the genes when compared to the Naïve group.

## 4. Discussion

TCDD is a known carcinogen and a well-characterized immunotoxic environmental contaminant [22]. Thus, it is beneficial to identify compounds that can neutralize the toxicity of TCDD. Previously, we showed that TCDD induced massive numbers of MDSCs when injected into mice [11]. Here, we provide evidence for resveratrol (RSV) to neutralize the effects of TCDD, including a significant reduction in MDSCs within the peritoneal cavity exudate (PEC) and altered differentiation of immune cell subsets in the peritoneal cavity (PC) at 24 h after TCDD administration. These data are consistent with the previous reports that RSV acts as an antagonist of AhR [23].

In a previous study, we reported that TCDD-mediated activation of AhR leads to the induction of large numbers of MDSCs that are primarily PMN-MDSCs and less of M-MDSCs [11], consistent with the current study. Interestingly, RSV inhibited TCDD-mediated induction of PMN-MDSCs but not M-MDSCs. While the reason for this is not clear, it is possible that TCDD induces many chemokines and cytokines that induce MDSCs, and RSV may not be able to block all of them. For example, we reported that TCDD induces chemokines and cytokines such as CCL2, CCL3, CCL4, CCL11, CXCL1, CXCL2, CXCL5, CXCL9, G-CSF, GM-CSF, VEGF, and M-CSF, and chemokine receptors on MDSCs such as CCR1, CCR5, and CXCR2. 

A current “gold standard” for MDSC evaluation is functional analyses of immunosuppressive activity via inhibition of T cell proliferation [11]. Interestingly, RSV reversed the immunosuppressive activity of MDSCs. This is important because MDSCs are well characterized for their role in cancer promotion by suppressing anti-tumor immunity [24]. Thus, if RSV can inhibit the induction and functions of MDSCs in a cancer model, it would be helpful in the treatment of cancer. 

Resveratrol promotes AhR translocation to the nucleus and binding to DNA at dioxin responsive elements, but subsequent transactivation does not take place [17]. Resveratrol inhibits the transactivation of several dioxin-inducible genes, including cytochrome P-450 1A1 and interleukin-1beta, both ex vivo and in vivo. Resveratrol has adequate potency and nontoxicity to warrant clinical testing as a prophylactic agent against aryl hydrocarbon-induced pathology.

In the current study, we also investigated the different types of immune cells that accumulate in the peritoneal cavity (PC) following TCDD administration. In addition to the induction of MDSCs, TCDD also had negative effects on cells that were F4/80+, a marker highly and constitutively expressed on resident macrophages [25]. Thus, TCDD decreased the proportion of macrophages while the TCDD+RSV group showed a marked increase in this population. Also, using CD11C, a marker primarily expressed by the dendritic cells and to a lesser extent M1 macrophages, TCDD was found to increase the percentage of these cells while the TCDD+RSV group showed a decrease. TCDD also caused a marked decrease in MHC Class-II-expressing cells, which are antigen-presenting cells including monocytes, macrophages, dendritic cells, and B cells, but not MDSCs [11,26], when compared to the vehicle. In contrast, the RSV+TCDD group showed slight recovery of such cells. Together, these studies suggest that TCDD can alter the proportions of myeloid cells and that RSV can reverse most of these effects. 

To better understand the gene expression profile of the MDSC populations from the various groups that we tested, we analyzed the transcriptome of Gr1^+^ MDSCs using microarray. We previously published work on the importance of C-X-C motif chemokine receptor 2 (Cxcr2) in our model, demonstrating that the treatment of mice with CXCR2 antagonist led to marked reduction in TCDD-induced MDSCs [9]. In the current study, we found that TCDD caused marked induction of CXCR2 while the TCDD+RSV group showed significant reduction in CXCR2 expression when compared to the TCDD group. This suggested that RSV may suppress TCDD-mediated MDSC induction by blocking CXCR2. We also found that RSV reversed the induction of several other inflammatory markers such as Myd88, CXCL1, and XBP1. MDSCs are well characterized for producing Arginase-1, which suppresses the T cell-mediated immune response [27]. This is one of the primary mechanisms through which MDSCs suppress anti-cancer immune response [27]. It is noteworthy that RES decreased the TCDD-mediated upregulation of Arginase-1, thereby suggesting that this may be the mechanism through which RSV reduces the immunosuppressive properties of TCDD-induced MDSCs, as seen in the current study. 

One interesting recent study demonstrated, using a transplanted tumor model, that RSV was able to block tumor growth by suppressing the induction of G-MDSCs, thereby permitting the anti-tumor CD8+ T-cells to act, as well as through the promotion of M-MDSC cells into mature CD11c+ dendritic cells or F4/80+ macrophages [28]. However, in this study, the role of AhR was not investigated. Thus, attenuation of the induction of MDSCs through use of RSV may help promote the anti-tumor immunity, thereby enabling better treatment of cancer, especially in cancer immunotherapy where MDSCs block the anti-tumor efficacy of the host [29].

Transcriptome changes related to metabolic processes were also highly altered in the TCDD+RSV group when compared to the TCDD group. Overall, TCDD increased the expression of metabolic process genes while treatment with RSV led to the downregulation of some of these genes. This was also corroborated by the observation that the TCDD+RSV group showed a decrease in the proton efflux rate during glycolysis. Together, this suggests the addition of RSV to TCDD mitigates the increased metabolic demands triggered by TCDD and alters gene expression related to myeloid function. 

In summary, the current study shows that RSV can prevent the potential immunosuppressive properties of TCDD by reversing the induction and functions of MDSCs. RSV may mediate these effects by suppressing some chemokines that are induced by TCDD as well as reversing the induction of Arginase-1, a highly immunosuppressive molecule present in MDSCs. The data support the processes related to metabolic function, which may also play an important role in the ability of RSV to reverse the effect of TCDD. In the clinical setting, MDSCs are considered to be the major obstacles to cancer immunotherapy because of their ability to reduce the anti-tumor T cell efficacy and cause tumor cells to become more resistant to immunotherapy. Thus, RSV holds significant promise in blocking MDSCs induced during tumor growth to promote anti-tumor immunity. Evidently, additional studies are necessary to study the clinical feasibility and efficacy of such an intervention.

## Data Availability

The datasets generated and/or analyzed during the current study are publicly available.

## Abbreviations

TCDD	2,3,7,8-Tetrachlorodibenzo-p-dioxin
RSV	3,4,5-trihydroxy-trans-stilbene; resveratrol
AhR	Aryl Hydrocarbon Receptor
MDSC	Myeloid-derived suppressor cell
PER	Proton efflux rate
LPM	Large peritoneal macrophage
SPM	Small peritoneal macrophage
PMN-MDSCs	Polymorphonuclear MDSCs
M-MDSCs	Monocytic MDSCs
NK	Natural Killer cells
NC	Neutrophils
DC	Dendritic cells
PI	Propidium Iodide
ConA	Concanavalin A
ALT	Alanine Aminotransferase
TUNEL	Terminal deoxynucleotidyl transferase dUTP nick end labeling
PC	Peritoneal Cavity
PEC	Peritoneal Cavity exudate
BM	Bone Marrow
CMC	Carboxymethyl cellulose
tSNE	t-distributed stochastic neighbor embedding
